# Measuring the quality of inpatient specialist consultation in the intensive care unit: Nursing and family experiences of communication

**DOI:** 10.1371/journal.pone.0214918

**Published:** 2019-04-11

**Authors:** Stephanie D. Roche, Alyse M. Reichheld, Nicholas Demosthenes, Anna C. Johansson, Michael D. Howell, Michael N. Cocchi, Bruce E. Landon, Jennifer P. Stevens

**Affiliations:** 1 Department of Health Care Quality, Beth Israel Deaconess Medical Center, Boston, Massachusetts, United States of America; 2 Department of Medicine, Beth Israel Deaconess Medical Center, Boston, Massachusetts, United States of America; 3 Department of Medicine, Harvard Medical School, Boston, Massachusetts, United States of America; 4 Center for Health Care Delivery Science and Innovation, University of Chicago Medicine, Chicago, Illinois, United States of America; 5 Department of Emergency Medicine, Harvard Medical School, Boston, Massachusetts, United States of America; 6 Department of Health Care Policy, Harvard Medical School, Boston, Massachusetts, United States of America; 7 Department of Pulmonary, Critical Care and Sleep Medicine, Beth Israel Deaconess Medical Center, Boston, Massachusetts, United States of America; 8 Center for Health Care Delivery Science, Beth Israel Deaconess Medical Center, Boston, Massachusetts, United States of America; University of Notre Dame Australia, AUSTRALIA

## Abstract

**Rationale:**

Critically ill patients in the intensive care unit (ICU) often require the care of specialist physicians for clinical or procedural expertise. The current state of communication between specialist physicians and families and nurses has not been explored.

**Objectives:**

To document the receipt of communication by nurses and family members regarding consultations performed on their patient or loved one, and to quantify how this impacts their overall perceptions of the quality of specialty care.

**Methods:**

Prospective survey of 60 adult family members and 90 nurses of 189 ICU patients who received a specialist consultation between March and October of 2015 in a single academic medical center in the United States. Surveys measured the prevalence of direct communication—defined as communication conducted in person, via telephone, or via text-page in which the specialist team gathered information about the patient from the nurse/family member and/or shared recommendations for care—and perceived quality of care.

**Results:**

In about two-thirds of family surveys (40/60) and one-half of nurse surveys (75/160), respondents had no direct communication with the specialist team that performed the consultation. Compared to nurses who had no direct communication with the specialists, those who did were 1.5 times more likely to rate the consultation as “excellent” (RR 1.48, 95% CI 1.2–1.8, p<0.001). Nearly 40% (22/60) of families knew so little about the consultation that they felt incapable of evaluating it.

**Conclusions:**

Most ICU families and nurses have no interaction with specialist providers. Nurses’ frequent exclusion from conversations about specialty care may pose safety risks and increase the likelihood of mixed messages for patients and families, most of whom desire some interaction with specialists. Future research is needed to identify effective mechanisms for information sharing that keep nurses and families aware of consultation requests, delivery, and outcomes without increasing the risk of mixed messages.

## Introduction

When inpatients require additional clinical expertise or procedures, specialist physicians may become temporarily involved in care through consultation.[1] Consultation is common in the intensive care unit (ICU) setting where critically ill patients often require specialist input. Although during consultation responsibility for patient care is not fully transferred to the specialist team, the consultation process is akin to a handoff in the sense that it involves substantial interclinician communication of patient-specific information critical to care continuity and patient safety.[2] In consultations as in handoffs, inadequate communication may jeopardize quality of care by contributing to adverse events, unnecessary care delays, and/or emotional harms to patients and families.[3] Communication is therefore a critical driver of consultative care quality[1].

Whereas communication during handoffs has been a major focus of patient safety research over the last decade—resulting in a diverse array of standardized checklists, forms, and training curricula[4–8]—communication during inpatient consultations has received comparatively less attention. To date, little is known about the current state of communication during consultations, including the flow, content, and timing of information exchange. Understanding what current communication within consultations looks like, and how its presence or absence may influence stakeholder perceptions of quality of care—is an important first step to developing quality metrics and improvement tools and strategies.

Most of the published literature on consultation quality has come in the form of specialty- and institution-specific guidelines,[9–11] general guidelines,[12–14] or opinion pieces offering advice [15–18] on how to conduct inpatient consultations. A handful of studies have surveyed physicians about their general communication preferences during consultation[19–21] or tested tools for improving trainee communication of consultation requests to specialists.[22,23] One major drawback to these publications, however, is that they predominantly describe what communication would look like in a hypothetical, ideal consultation, rather than providing insight into what communication actually looks like in real consultations. A second drawback is that they narrowly focus on physician-to-physician communication to the exclusion of communication with nurses, patients, family members, or other members of the care team. Lastly, they are not specific to the ICU setting, a care environment in which carrying out consultations—and managing information about them—may pose unique challenges that do not manifest in the same way on the floor or in the outpatient setting.

In this study, we sought to (1) document the receipt of communication by ICU nurses and family members regarding consultations performed on their patient or loved one, and (2) quantify how the presence or absence of direct communication with the specialist impacts these stakeholders’ perceptions of the quality of specialty care. Although stakeholder perceptions of care quality are less objective than other measures (such as infection rates or staffing compliance), they still merit consideration as they can potentially reflect the downstream effects of deficits in care quality, identify important components of quality not currently measured, and/or provide insight into patient-centeredness—one of the six domains of care quality.[24] We chose to focus specifically on specialist communication because, during their temporary involvement in care, specialists represent a new source of information for nurses and families. As external physicians brought in for their expertise, specialists are uniquely positioned to shape nurse and family member understanding of and/or confidence in the care plan. We hypothesized that specialists rarely interact with nurses and family members when delivering consultative care in the ICU, and that a lack of direct communication may negatively impact how nurses and family members perceive the quality of consultative care.

## Methods

### Study design and setting

Between March and October of 2015, we implemented a prospective survey of adult family members and nurses of 200 adult ICU patients who received a specialty physician consultation in one of four adult ICUs at a single academic medical center: two medical ICUs, each with 8 beds; a medical-surgical ICU with 12 beds, and a surgical ICU with 15 beds. All consultations completed within the previous 48 hours were eligible for inclusion in our study so long as the patient was still an inpatient at the time the consultation was identified. Consultations provided by non-physicians, such as nutrition, speech and language pathology, and spiritual care, were excluded. Surveys were completed an average of 1 day after the consultation occurred (SD 2.3 days).

### Participants

The patient’s primary nurse who documented care in the electronic medical record at the time of the consultation and an adult family member or friend of the patient were eligible to participate, with preference given to the patient’s health care proxy and/or next-of-kin. All family members had to report comfort with speaking English during the consent process. Family members were excluded in cases where the patient died prior to the family member being invited to participate. For all consultations for which we received at least one completed survey, we extracted patient information from the medical record.

### Survey instrument

In the absence of a validated tool to capture stakeholder experiences of consultation communication and perceptions of consultation quality, we developed a novel survey (S1 and S2 Tables), derived from our prior qualitative work on consultation in the ICU.[1] One author (JPS) developed the survey, and an expert in survey data analysis (BEL) and an investigator with graduate training in survey research (SDR) assisted with iterative improvements. The surveys were administered using REDCap (Research Electronic Data Capture), a secure web-based application.[25] Using cognitive interviewing techniques,[26] we pretested the survey instrument with critical care nurses and former ICU patients/family members who were part of the medical center’s ICU Patient and Family Advisory Council. Questions were iteratively improved for clarity and feasibility until no additional recommendations were made[27].

The surveys took 3–5 minutes to complete and used closed-ended questions to elicit details about the consultation. With respect to communication timing, our survey captured whether the respondent knew about the consultation prior to it occurring, and with respect to communication content, our survey captured whether the specialist spoke with the respondent—in person, via telephone, or via text page—to gather information about the patient and/or share recommendations for care. Rather than asking family members to rate overall quality, which might be difficult for them to assess, they were asked to rate the overall timeliness of consultation and quality of communication. Nurses were asked to rate the consultation’s urgency, impact, and overall quality. In both surveys, an answer choice of “I don’t know/remember” was available for all non-demographic questions. The surveys collected limited demographic data and included a free-text box where participants could add additional feedback, which was subsequently analyzed as qualitative data.

The exposure of interest—a binary description of the type of communication with the specialist team—was determined prior to analysis. Participants with whom the specialist team spoke in person, via telephone, or via text-page to gather information about the patient and/or share recommendations were defined as having “direct communication”. Those who did not have any communication with the specialist team were defined as having “no direct communication”.

The primary outcome of interest among the family cohort was timeliness of the consultation, evaluated as part of the survey instrument on a 5-point scale with 5 anchored as “excellent” and 1 as “terrible”. The primary outcome of interest among the nursing cohort was overall rating of the quality of the consultation, anchored on the same 5-point scale. Given the ceiling effect noted in the results, both variables were collapsed to “excellent” and “less than excellent” in multivariate analyses.

Covariates of interest collected included patient gender and age, ICU admission and discharge dates, length of stay, consultation service, and measures of the patient’s severity of illness using the Sequential Organ Failure Assessment (SOFA) score on the day of ICU admission and the day of consultation.[28] Covariates collected from nurses included gender, age category, and number of years working at the medical center. Covariates collected from families included gender, age category, relationship to the patient, and whether the family member lived in the same metropolitan area as the hospital, information previously associated with family member satisfaction of care in the ICU.[29] Admission-level variables collected included campus (out of two total campuses) on which the consultation occurred and whether the consultation took place on a weekend.

### Data collection

On weekdays, trained research assistants (RAs) visited two ICUs per day so that each of the four ICUs received two visits per week. The order of visits varied so that consultations occurring on weekends would be captured on Monday or Tuesday of the following week. Between the hours of 9am and 1pm, research assistants entered the ICU and spoke with the responsible physician and/or fellow to identify new consultations requested for patients currently in the unit within the previous 48 hours. Once the consultation occurred, RAs identified the responsible physician and nurse of record at the time the consultation occurred and administered surveys until 6pm. For consultations that occurred over the weekend, the name of the primary nurse on shift at the time of the consultation was abstracted from the medical record on Monday or Tuesday of the following week. If the nurse happened to be present on the day of survey administration, s/he was invited to participate in person with the survey available on an electronic tablet. Otherwise, s/he received up to two e-mail invitations, which included the study introduction script, consent, and a direct link to the survey. Eligible family members were invited to participate in the study either in the patient’s room or in the ICU family waiting room. Due to institutional review board (IRB) restrictions, family member surveys were only collected in person.

### Statistical analysis

All statistical tests were performed using SAS 9.3 (SAS Institute, Cary, NC). The unit of analysis was the individual consultation. Descriptive statistics of the data were performed, including description of central tendency and variance as mean and standard deviation. We used Pearson’s chi-square test and the student’s t-test to compare distributions among populations as appropriate. Multivariable analyses were performed using generalized estimating equations. Individual nurses completed an average of 1.8 surveys, though never on the same patient more than once. To control for the effect of greater participation among some nurses, we clustered consultations by nurse in our multivariable analyses. Ten patients were included in the study twice because they had two separate ICU admissions during the study period, but no more than one family and one nurse survey was collected for each of these ten patients. Variables were selected for inclusion if they had a p-value of 0.05 or less. One consultation for which no participants completed a survey was excluded from our study. Participants who selected “I don’t know/remember” were excluded from relevant analyses. For example, nurses who selected “I do not know/remember” for the question about overall quality of the consultation were excluded from the regression of direct communication on quality rating. The de-identified dataset is available from the Harvard Dataverse repository at https://doi.org/10.7910/DVN/JDJBSR.

### Qualitative analysis

Qualitative, free-text data that nurses provided about the consultation in the survey’s open-ended question was analyzed using conventional content analysis.[30] Free-text comments were collated into a single document and reviewed multiple times in their entirety to achieve an overall understanding of the data. Preliminary codes representing distinct concepts were then identified and applied to a sample of the free-text data. Codes were refined iteratively, with some broadened to capture all facets of the same concept and others narrowed to further distinguish between two concepts. The final codes were applied to the full dataset and paired with representative quotes.

### Research ethics

This study was approved by the IRB of Beth Israel Deaconess Medical Center (Committee on Clinical Investigations approval #2015P-000043). Informed consent was obtained electronically by all participants. Participants were presented with an IRB-approved consent script and had to click an “I ACCEPT” button in order to access the survey. The funding agencies played no role in the design and conduct of the study; the collection, management, analysis, and interpretation of the data; or the preparation, review, or approval of the manuscript.

## Results

### Patients and participants

**Fig 1:**
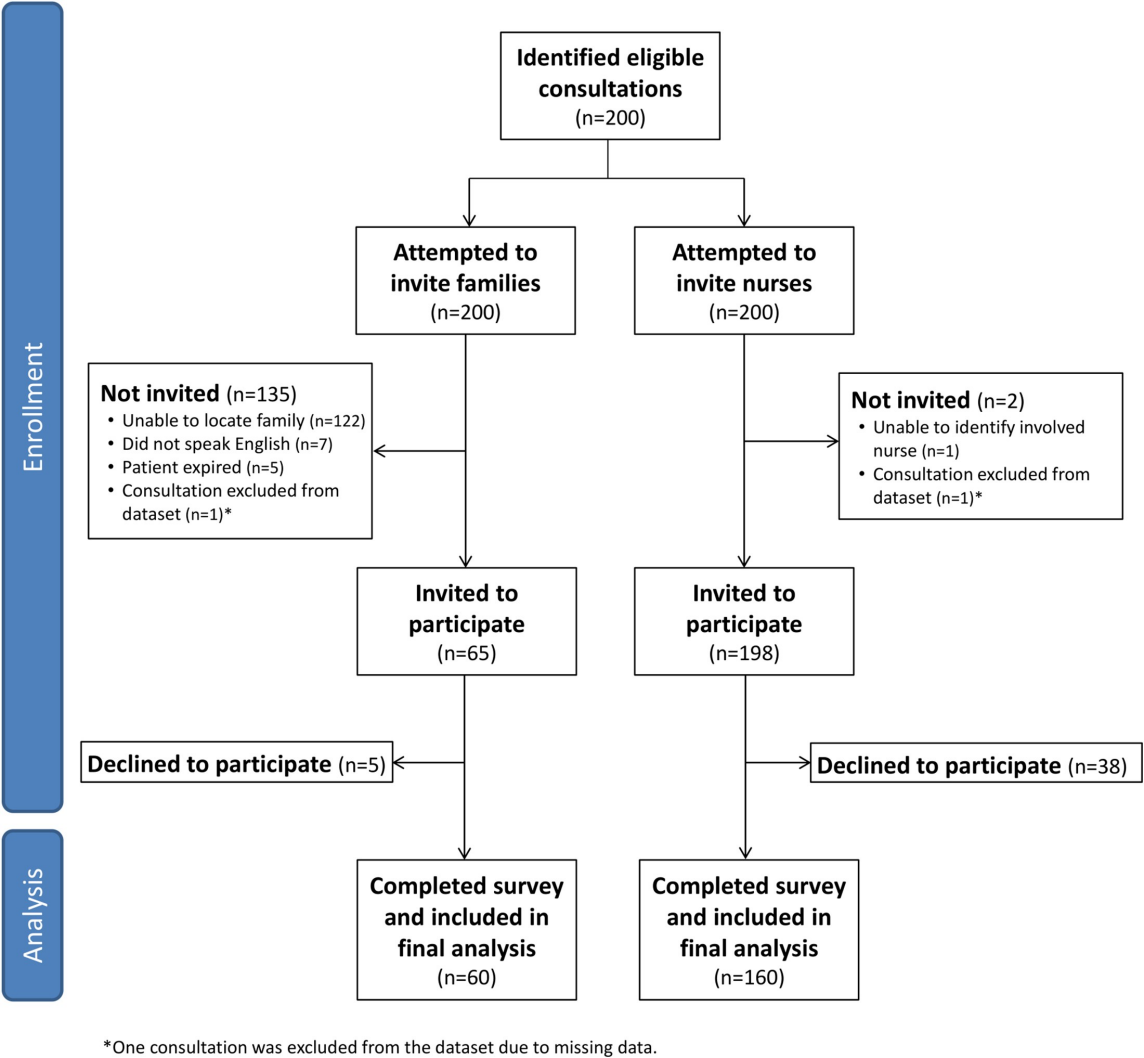
Flow diagram depicting study population.

199 consultations involving 189 unique patients were evaluated in our study. Of these consultations, we could not locate a family member for 122, the family participant did not speak English for 7, and the patient died prior to survey administration for 5. Of the remaining 65 consultations, 60 family members agreed to participate (overall response rate 30%, response rate among those approached, 92%) and 90 nurses participated for 160 consultations (80% response rate) (Consort diagram, Fig 1). Patient characteristics are summarized in Table 1. Family and nurse characteristics are described below and summarized in S3 Table. Both family and nurse participants were majority female (65% and 94%, respectively), and patients were majority male (61%). On average, nurses were 40 years old and had 12 years of work experience at the medical center; 81% of family members were between the ages of 40 and 79. Two-thirds of families (40/60) did not live in the same metropolitan area as the medical center, and about three-fifths (37/60) were either the patient’s spouse or son/daughter. The most common type of specialist consultation was nephrology (11%, 21/199), followed by gastroenterology (10%, 20/199), cardiology (9%, 18/199), and general surgery (9%, 18/199). Patient average length of stay was 17 days.

**Table 1:** Patient demographics*.

	Patients (n = 189)**
**Female**	74 (39.2%)
**Age category, years**	
18 to 3918 to 39	16 (8.5%)
40 to 59	49 (25.9%)
60 to 79	97 (51.3%)
80 to 99	27 (14.3%)
**Mean SOFA score on day of ICU admission**	8.0 (4.0)
**Mean SOFA score on day of consultation**	8.1 (4.4)
**Top 3 medical consultations**	
Nephrology	21 (10.6%)
GI	20 (10.1%)
Cardiology	18 (9.0%)
**Top 3 surgical consultations**	
Surgery	18 (9.0%)
Urology	4 (2.0%)
ENT	4 (2.0%)
**LOS category, days**	
5 or fewer	33 (16.6%)
6 to 10	50 (25.1%)
11 to 15	30 (15.1%)
More than 15	86 (43.2%)

*Numbers are presented as n(%) or mean(SD) depending on variable type.

**Proportions for gender and age category variables are out of a denominator of 189 so as not to double-count these individuals. All other variables are out of a denominator of 199 because they refer to the individual consultation.

### Communication around the involvement of a specialist in patient care

Overall, family members had direct communication with the specialist team 33% (20/60) of the time (Fig 1). Out of these 20 consultations, the specialist gathered information about the patient from the family in 4, shared recommendations with the family in 5, and did both in 11. When asked if they knew about the consultation prior to it occurring, about half of families (53%, 32/60) said yes. Among these families, 8 had requested the consultation. Most of the remaining families learned about the consultation request from a physician member of the primary team (34%, 11/32) or the nurse (22%, 7/32).

**Fig 2:**
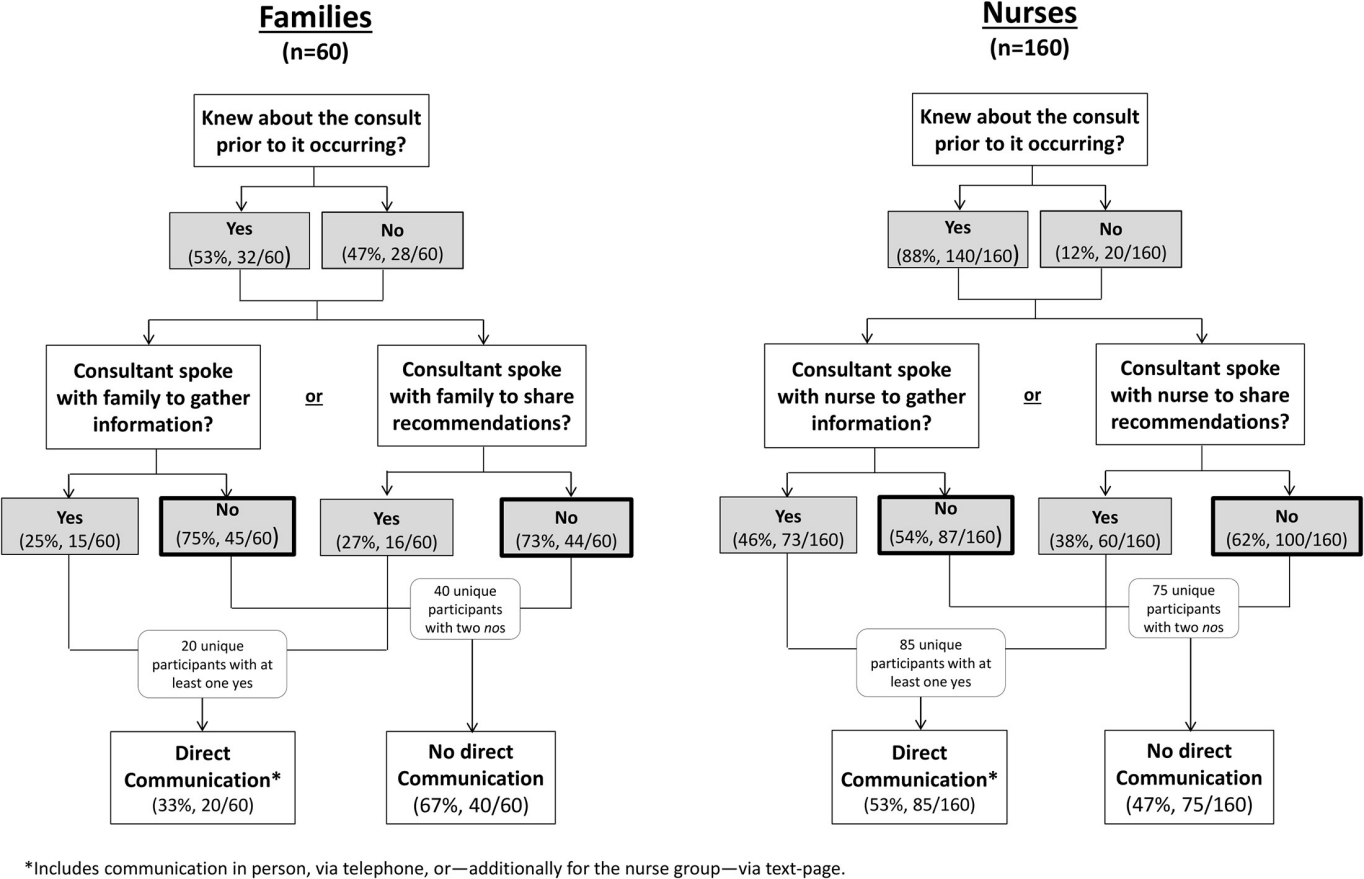
Definition and frequency of communication experienced by study participants.

The specialists communicated with nurses directly 53% (85/160) of the time (Fig 2). Of these 85 consultations, the specialist gathered information about the patient from the nurse in 25, shared recommendations with the nurse in 12, and did both in 48. Eighty-eight percent (140/160) of nurses had knowledge of the consultation before it happened. Of these 140 nurses, nine (6%) requested the consult themselves, 90 (56%) learned about the consultation from a physician member of the primary team, 14 (10%) from a fellow nurse, 11 (8%) from participation in morning rounds, 6 (4%) from reading the specialist’s note in the patient’s medical record. Family and nurse characteristics were not significantly related to direct communication with the specialist (S4 Table).

### Family preferences for communication

When asked with whom they most prefer to talk about how their family member is doing, families most frequently identified the specialist team (30%, 18/60), the head ICU doctor (30%, 18/60), or the nurse (20%, 12/60). When asked with whom they prefer to talk when they want to know the specialist’s thoughts on the patient’s diagnosis and next steps for care, most families (63%, 38/60) said the specialist team, followed by the nurse (15%, 9/60) and the head ICU doctor (12%, 7/60).

### Contribution of communication to evaluation of consultation quality

#### Family ratings of the consultation

Twenty-two families (37%, 22/60) felt they were unable to rate the timeliness of specialist involvement because of their lack of knowledge about the consultation. Of the 38 families who did offer an assessment, most rated the timeliness “excellent” (74%, 28/38) or “good” (21%, 8/38). Only one family each evaluated the timeliness as “okay” or “bad”. Compared to respondents who were the patient’s sibling, parent, or other relative, a greater proportion of respondents who were the spouse or child of the patient rated the consultation’s timeliness as “excellent” (p-value = 0.005, S5 Table).

Most families (67%, 40/60) did not meet the specialist team and therefore could not rate the quality of communication; however, among those families that did have such interaction, most rated the communication quality “excellent” (70%, 14/20) or “good” (25%, 5/20). Only one rated it as “okay”.

#### Nurse ratings of the consultation

**Fig 3:**
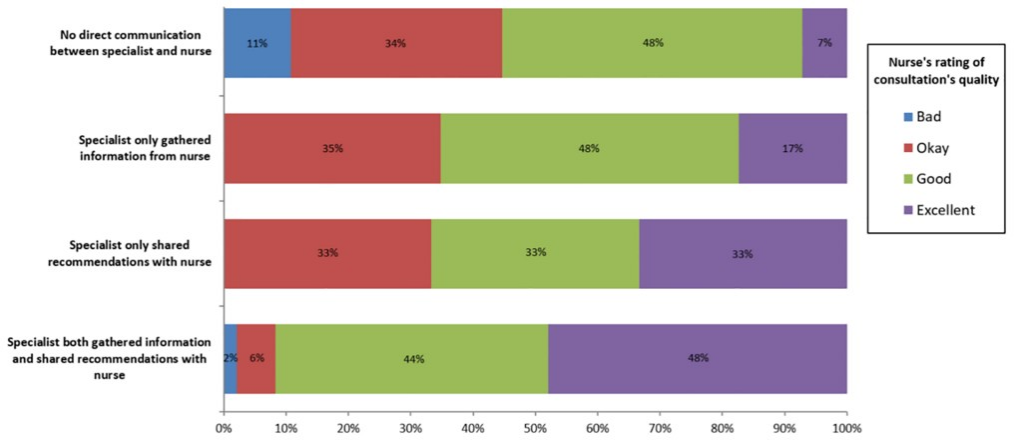
Breakdown of nurses’ quality ratings by level of direct communication with specialist team.

Nurses described most consultations as either “excellent” (22%, 35/160) or “good” (39%, 63/160) while 4% rated the consultation as “bad” (7/160). As direct communication between the nurse and specialist increased, a greater proportion of nurses rated the consultation’s quality as “excellent” (Fig 3).

Compared to nurses that had no direct communication with the specialist, nurses that received any type of direct communication were 1.5 times more likely to rate the consultation as “excellent” (RR 1.48, 95% CI 1.2–1.8, p < .0001) and 5.5 times more likely to report that the consultation added value to the patient’s care, although this latter effect estimate should be interpreted with caution as its confidence interval indicates that it may be as low as 1.2 (RR 5.49, 95% CI 1.2–25.4, p = .01).

After adjustment for campus of the hospital, having direct communication with the specialist team increased the odds that the nurse would perceive the consultation to be of excellent quality by more than seven-fold (OR 7.4, 95% CI 2.3–24.0, Table 2). This effect was not surprising because the hospital’s two campuses are one-half mile apart in distance and separated by a busy intersection; therefore, if the specialty service being consulted is located on the opposite campus as the requesting ICU, then the consultation may take longer to occur. However, despite achieving statistical significance, this effect estimate is characterized by substantial uncertainty, as indicated by its wide confidence interval.

**Table 2:** Odds ratio estimates of GEE with Logit link, clustered by nurse*.

Effect	Odds Ratio	95% Wald Confidence Limits	
Direct vs. no direct communication	7.860	2.535	24.364
East vs. west campus	3.222	1.312	7.914

*Modeling nurse perception of “excellent” consultation quality. Interaction between type of communication and campus was tested but was not significant (p = 0.41).

### Qualitative themes of nurse-specialist interaction

Forty-four nurses offered free-text feedback on the consultation, and analysis revealed two common themes (Table 3). The first theme was nurses crediting their knowledge of the consultation to their self-initiative to solicit information from the specialist. These nurses described scenarios in which the specialist initially did not freely offer information to the nurse but shared it once the nurse requested it. The second theme was the importance for nurses to know consultation information, both for patient care and for delivering a consistent message to the patient/family.

**Table 3:** Sample quotes from feedback in nurse surveys.

Theme	Sample Positive or Neutral Quotes	Sample Negative Quotes
Nurses take the initiative to solicit consultation information from the specialist.	“I had to approach the [specialist] team for info and follow-up.”	“I had to ask [the specialist] specific questions about why the consultation was taking place and [about the] findings or recommendations, and I happened to see [the specialist] going into the room, so I followed her. No information was freely offered.”
		“[The specialist] discussed taking the patient to surgery. If I had not asked him, I do not believe he planned on telling me he was taking my patient to surgery today.”
Importance for nurses to know consultation information, as this can impact patient care, unit workflow, and communication with the patient/family.	“[The specialist] was good about letting a staff member know that pupils would be dilated, which was very important to the nurse doing Q1hr [every hour] neuro[logical] assessment.”	“[The specialist team] may have spoken with the neurosurgery team about their recommendations, but didn’t touch base with the nursing staff. That would have been helpful since they probably dilated his eyes, which is good to know for a neuro[logical] patient.”
	“If it was decided that the patient did need CRRT [continuous renal replacement therapy], it would have a huge impact on nursing and staffing, particularly when staffing was short…CRRT patients are always singled [one-to-one nurse-to-patient ratio], and it would have been very difficult to rearrange the [patient] assignment [to nurses]. So the more advanced notice and communication from [the specialist], the more efficient the treatment and possibly the patient outcome.”	“This patient and family were very anxious about everything…One word was different between the [specialist team], me, and the [primary] team, which made the patient’s wife fairly distressed for part of the day.”

## Discussion

To our knowledge, this study is the first to explore how specialists interact with families and nurses during the inpatient specialty consultation process. Our findings reveal that communication about consultations in the ICU is often fragmented, leaving families and nurses uninformed about the latest developments in the care plan. Even when information is shared, nurses and families receive variable amounts of it, primarily second-hand, and in unpredictable patterns, in contrast with stated preferences. Our study indicates that, when it comes to specialty consultation, information is not frequently solicited from family members and nurses by the specialist team.

Nurses in our study who received direct communication from the specialist had only slightly higher odds of rating the consultation quality as “excellent” compared to their counterparts. One potential reason for this small effect estimate is that when nurses rate the overall consultation quality, they may consider other factors not captured by our exposure variable, such as the clinical relevance of the consultation.[1] For example, it is possible that nurses value direct communication more for procedural consultations than for cognitive ones since the former are likely to affect the nurse’s own workflow to a greater extent than the latter. The effect of direct communication on the odds of nurses reporting that the consultation added value to care was characterized by considerable uncertainty, resulting in a wide confidence interval. Again, this effect estimate may be confounded by factors not captured in our model. Further research is needed to understand what factors these stakeholders take into consideration when asked for their perceptions of care quality, and to explore the relationship between quality perceptions and other exposures beyond direct communication with the specialist.

Although we found direct communication to have only a marginal impact on stakeholder perceptions of consultation care quality, this does not mean its presence/absence, content, and/or timing does not impact other components of care quality, such as safety. Nurses in our study explicitly identified examples where communication with specialists about bedside interventions (e.g., dilating a patient’s eyes for an ophthalmological exam) was critical to avoiding urgent and unnecessary additional studies. Inconsistent knowledge among the care team about consultations may also increase the likelihood that patients and families receive discordant messages about the care plan, which can cause them unnecessary distress.[31–34] Lastly, non-engagement of ICU families by specialists ignores that families often act as surrogate decision makers, advocate for the patient’s care preferences, and relay critical information between patients and clinicians.[35–39] When critically ill patients are incapacitated to make autonomous decisions—as 95% of critically ill adults are estimated to be [40]—specialists may play a key role in the shared-decision making process by providing surrogates with clinical expertise and helping them weigh the different care options in light of the clinical evidence, anticipated benefits and risks, and care preferences.[41] Specialist communication may be especially valued for understanding the potential benefit of high-risk, invasive procedures such as deciding whether to initiate renal replacement therapy in the setting of acute kidney injury, or the likelihood of neurologic recovery in the setting of devastating neurologic injury.

However, securing direct communication between specialists and ICU nurses and family members during consultations is fraught with logistical challenges. First, whereas for many healthcare processes involving communication, information exchange generally occurs at a discrete point in time (e.g., upon admission or discharge), a consultation—especially one for a critically ill patient—may span multiple shifts, with the specialist team following the patient over several days or weeks. The longer a consultation lasts, the more times information needs to be exchanged to different individuals involved in the patient’s care. Second, the content of the consultation information for a critically ill patient will likely change over time, as these patients are prone to abrupt status changes. Initial recommendations offered by the specialist may evolve as new information about the patient becomes available. In that scenario, the consultation information needs to be updated regularly. Third, whereas nurses and physicians on the primary team work together regularly, specialists are typically external to the immediate care team. Lack of familiarity between the specialist and nurses may hinder direct communication between them. Lastly, specialists provide care for patients at different times throughout the day and night rather than in scheduled rounds, thereby making it difficult for nurses and family members to be present when the consultation occurs or when the specialists are ready to share their recommendations. Yet, nurses and family members sometimes know important information that could influence the specialist’s assessments. For example, family members acting as surrogate decisionmakers may know ahead of time that they will decline a particular specialist intervention if it is proposed. If this information is solicited by the specialist during the consultation, then it can inform his or her recommendations for care. Absent that communication, the specialist may formulate recommendations that are bound for rejection.

In addition to these logistical challenges, there are many other aspects of ICU consultation communication that merit further research. For example, we currently lack knowledge regarding whether there are any circumstances under which indirect communication might be acceptable or even preferred. The qualitative data from our study suggests that most nurses’ dissatisfaction was primarily rooted in their overall lack of knowledge about the consultation; it is possible, however, that nurses’ perceptions of quality would improve if they were kept up to date through non-direct means, such as through the primary team’s head physician. For patients and families, their desire for direct communication with specialists must be balanced against the need for a unified message from specialists and the primary team about the care plan, as it is possible that increasing direct communication between specialists and families could confuse families, especially if specialists use different terminology than the primary team. More research is needed to identify the frequency, content, and method of communication with specialists that would meet the informational needs of nurses. The same assessment should also be done for families. Once nurse and family informational needs are understood, tools to address each group’s needs—such as a consultation checklist or family-facing web portal[42,43] for tracking and facilitating communication—should be tested, with particular attention paid to whether these tools can improve communication across different shifts and settings. Numerous communication tools and interventions have been developed for improving communication in general among ICU primary team members and between the ICU primary team and patients/families. These include provider training on team communication and conflict resolution,[44] daily goals worksheets,[45] use of trained communication facilitators,[46] implementation of bedside and family rounds,[47] and structured approaches for conducting and documenting family meetings.[48] Additional research is needed to determine whether these interventions, or adaptations of them, might be effective in the context of ICU consultations. Because non-English speaking families may have even more barriers to obtaining accurate communication about the care plan, further research is needed on this population. Lastly, future studies could explore the impact of communication deficits on patient- and family-level outcomes, including measures of emotional harm such as stress and depression.

### Study strengths and limitations

The main strengths of this study are its inclusion of multi-stakeholder perspectives and involvement of ICU patients, families, and nurses in cognitive testing, the use of a survey instrument based on a formal qualitative conceptual model, and the collection of data in as close to real time as feasible. The main limitation of this research is that it was a single center study restricted to English-speaking ICU families and nurses. While we believe that the acuity of patients in ICU settings makes inpatient specialty care that much more important, this may limit the generalizability of our findings to other practice settings, regions, and non-English-speaking populations. Family members who were present and able to participate may be meaningfully different than family members who were not present, suggesting our family survey results are prone to selection bias. The small number of family members who received an invitation to participate in the study and agreed to participate (n = 60) limited our statistical power to detect differences in quality ratings by factors such as type of communication received. Because we only surveyed the primary nurse who was on shift when the consultation took place, we did not capture communication that may have occurred between the consulting team and the nurse during previous shifts. Also, because nurses and families were not surveyed immediately following the completion of the consultation, it is possible that some did not accurately remember the communication they had with the specialist. To minimize recall bias, we surveyed participants as soon after the consultation as feasible, with most completing the survey within 24 hours of the consultation’s completion. We also reminded participants of the consultation type, when it occurred, and the name of the specialist during the study invitation and consent process. In regression analyses, we excluded participants who answered “I don’t know/remember” to the questions of interest. The ceiling effect noted in the nurse quality ratings suggests that an alternative scale may assess perceptions of quality more precisely. Developing such a scale is an area for future study. Lastly, we suspect that communication between nurses and specialists may vary by the type of specialty care provided. Our study was not designed to detect a difference in communication by consultation type, but this topic merits investigation in future research.

## Conclusion

Current communication flows related to inpatient specialty care in the ICU do not follow predictable patterns. Families often learn about specialist consultations after they have already occurred, and both families and nurses frequently do not have any direct communication with the specialist team. Future research is needed to identify effective mechanisms for information sharing to keep nurses and family members aware of consultation requests, delivery, and outcomes.

## Supporting information

S1 Table Family survey. (DOCX)

S2 Table Nurse survey. (DOCX)

S3 Table Family and nurse demographics. (DOCX)

S4 Table Univariate associations of patient and participant features, divided by participants’ communication with subspecialist team. (DOCX)

S5 Table Univariate associations of patient and participant features, divided by participants’ rating of the consultation’s quality. (DOCX)
