# Supplementary material for: Measuring the quality of inpatient specialist consultation in the intensive care unit: Nursing and family experiences of communication
Source: PLoS One. 2019 Apr 11;14(4):e0214918. doi: 10.1371/journal.pone.0214918 (PMC6459595; doi:10.1371/journal.pone.0214918)
Supplement: S1 Table — (DOCX) [file pone.0214918.s001.docx]

Data Supplement for

*“Measuring the quality of inpatient specialist consultation in the intensive care unit: Nursing and family experiences of communication”*

Stephanie D. Roche, Alyse M. Reichheld, Nicholas Demosthenes, Anna C. Johansson, Michael D. Howell, Michael N. Cocchi, Bruce E. Landon, Jennifer P. Stevens

The de-identified dataset is available from the Harvard Dataverse repository at <https://doi.org/10.7910/DVN/JDJBSR>.

**S1 Table. Family Survey**

|  | **Question** | **Answer Options** | **Skip Patterns** |
| --- | --- | --- | --- |
| 1 | What is your relationship to the patient? | -Parent  -Spouse  -Son/Daughter  -Friend  -Other: _________________ |  |
| 2 | What is your age? | [free-text box] |  |
| 3 | What is your gender? | -Male  -Female |  |
| 4 | Do you live in the Boston metropolitan area? | -Yes  -No |  |
| 5 | Were you aware that the ICU team talked to a specialist about your family member (the patient) today? | -Yes  -No | “No” skips to question #11 |
| 6 | Did you or another family member request that a specialist be called in? | -Yes  -No | “Yes” skips to question #9 |
| 7 | Before the specialist came, did someone on the ICU team let you know that a specialist was going to become involved in your family member’s case? | -Yes  - No  - I do not know/remember | “No” skips to question #9 |
| 8 | Who told you that a specialist was going to become involved in your family member’s case? | -A member of the ICU Team  -The nurse  -Other: _______________  - I do not know/remember |  |
| 9 | Did the specialist speak with you or another family member to gather more information about your family member (the patient)?  *(For example, information about the patient’s medical history or how s/he came to the hospital)* | -Yes  -No  - I do not know/remember |  |
| 10 | Did the specialist speak with you or another family member to share his or her recommendations for your family member’s care?  *(For example, thoughts about the diagnosis or next steps to be taken)* | -Yes  -No  -I do not know/remember |  |
| 11 | Who do you most prefer to talk with about how your family member is doing? | -The specialist team  -The ICU attending  -Other physician members of the ICU team  -The nursing members of the ICU team  -Other: ______________  -I do not know |  |
| 12 | When you want to know about the specialist’s thoughts on your family member’s diagnosis and next steps for care, who do you most prefer to talk with? | -The specialist team  -The ICU attending  -Other physician members of the ICU team  -The nursing members of the ICU team  -Other: ______________  -I do not know |  |
| Please rate the following features of this consult on a 5-point scale, where 5 is “excellent” and 1 is “terrible”, with reference to the consult as a whole. | | | |
| 13 | Timeliness of consultation  *(For example, how long it took the specialist to arrive and give his/her opinion or perform needed services)* | 5 – Excellent  4 – Good  3 – Okay  2 – Bad  1 – Terrible  I do not know |  |
| 14 | Communication between the specialist team and you/your family member(s), including the patient  *(For example, when the specialist explained what the next steps would be)* | 5 – Excellent  4 – Good  3 – Okay  2 – Bad  1 – Terrible  I do not know |  |
| 15 | Communication between the ICU team and you/your family member(s), including the patient  *(For example, when the ICU team explained what the next steps would be)* | 5 – Excellent  4 – Good  3 – Okay  2 – Bad  1 – Terrible  I do not know |  |
| 16 | Do you have any other general or specific feedback that you would like to offer? | [free-text] |  |
