# Supplementary material for: Measuring the quality of inpatient specialist consultation in the intensive care unit: Nursing and family experiences of communication
Source: PLoS One. 2019 Apr 11;14(4):e0214918. doi: 10.1371/journal.pone.0214918 (PMC6459595; doi:10.1371/journal.pone.0214918)
Supplement: S2 Table — (DOCX) [file pone.0214918.s002.docx]

Data Supplement for

*“Measuring the quality of inpatient specialist consultation in the intensive care unit: Nursing and family experiences of communication”*

Stephanie D. Roche, Alyse M. Reichheld, Nicholas Demosthenes, Anna C. Johansson, Michael D. Howell, Michael N. Cocchi, Bruce E. Landon, Jennifer P. Stevens

The de-identified dataset is available from the Harvard Dataverse repository at <https://doi.org/10.7910/DVN/JDJBSR>.

**S2 Table. Nurse survey**

|  | | **Question** | **Answer Options** | **Skip Patterns** |
| --- | --- | --- | --- | --- |
| 1 | | What is your age? | [free-text box] |  |
| 2 | | What is your gender? | -Male  -Female |  |
| 3 | | Including this one, on how many shifts have you cared for this patient? | [free-text box] |  |
| 4 | | How many years have you worked at Beth Israel Deaconess Medical Center in any role? | [free-text box] |  |
| 5 | | Before the consultation occurred, did you know a consultation was called on your patient? | -Yes  -No | “No” skips to question #7 |
| 6 | | Did you request the consultation for the patient? | -Yes  -No | “Yes” skips to question #8 |
| 7 | | How did you find out that a consultant would become involved? | -When the consultation saw the patient  -A member of the ICU team  -A fellow nurse  -Another consultant in the unit at the time  -The patient  -I saw it in the patient’s medical record  -Other: ______________  - I do not know/remember |  |
| 8 | | Did the consultant speak with you to gather more information about the patient?  *(For example, did the consultant ask you for information about the patient’s medical history or presentation to the hospital?)* | -Yes  -No |  |
| 9 | | Did the consultant speak with you to communicate his or her recommendations?  *(For example, did the consultant talk to you about the patient’s diagnosis or next steps for care?)* | -Yes  - No  - I do not know/remember |  |
| 10 | | Did the consultant speak to the family about the plan of care? | -Yes  -No  -I do not know/remember | “No” skips to question #12 |
| 11 | | Did the plan of care communicated to the family by the consulting team match the plan of care communicated to the family by the ICU team? | -Yes  -No  - I do not know/remember |  |
| 12 | | In your opinion, did this consultation add value to the overall care of the patient? | -Yes  -No  - I do not know/remember | “No” and “I don’t know/remember” skips to question #14 |
| 13 | | In your opinion, how much of an impact did the consultation have in the care of the patient?  *(This may include impact on the patient’s clinical management, impact on the quality of communication, or impact on the overall value added by the consult.)* | -Very high impact  -Moderate impact  -Low impact  -Very low (near-negligible) impact |  |
| 14 | | In your opinion, how urgent was the consultation? | -Very urgent: The patient needed the consult immediately (within the hour)  -Somewhat urgent: The patient needed the consult soon (in the next four hours)  -Not very urgent: The patient needed the consult before the end of the day  -Non-urgent: The patient needed the consult by tomorrow.  -I do not know/remember |  |
| Please rate the following features of this consult on a 5-point scale, where 5 is “excellent” and 1 is “terrible”, with reference to the consult as a whole. | | | | |
| 15 | Quality of the communication between the consultant team and the physician members of the ICU team | | 5 – Excellent  4 – Good  3 – Okay  2 – Bad  1 – Terrible  I do not know |  |
| 16 | Quality of the communication between the consultant team and the nursing members of the ICU team | | 5 – Excellent  4 – Good  3 – Okay  2 – Bad  1 – Terrible  I do not know |  |
| 17 | Quality of the communication between the consultant team and the patient/patient’s family | | 5 – Excellent  4 – Good  3 – Okay  2 – Bad  1 – Terrible  I do not know |  |
| 18 | Overall quality of the consult | | 5 – Excellent  4 – Good  3 – Okay  2 – Bad  1 – Terrible  I do not know |  |
| 19 | Do you have any other general or specific feedback that you would like to offer about this or other consultations in which you’ve been involved? | | [free-text] |  |
