# Supplementary material for: Measuring the quality of inpatient specialist consultation in the intensive care unit: Nursing and family experiences of communication
Source: PLoS One. 2019 Apr 11;14(4):e0214918. doi: 10.1371/journal.pone.0214918 (PMC6459595; doi:10.1371/journal.pone.0214918)
Supplement: S3 Table — (DOCX) [file pone.0214918.s003.docx]

Data Supplement for

*“Measuring the quality of inpatient specialist consultation in the intensive care unit: Nursing and family experiences of communication”*

Stephanie D. Roche, Alyse M. Reichheld, Nicholas Demosthenes, Anna C. Johansson, Michael D. Howell, Michael N. Cocchi, Bruce E. Landon, Jennifer P. Stevens

The de-identified dataset is available from the Harvard Dataverse repository at <https://doi.org/10.7910/DVN/JDJBSR>.

**S3 Table. Family and nurse demographics***

|  | **Families**  **(n=60)** | **Nurses**  **(n=90)** |
| --- | --- | --- |
| **Female** | 39 (65.0%) | 85 (94.4%) |
| **Age category, years**  **18 to 39** | 10 (16.7%) | 49 (55.7%) |
| **40 to 59** | 31 (51.8%) | 35 (39.8%) |
| **60 to 79** | 18 (30.0%) | 4 (4.6%) |
| **80 to 99** | 1 (1.7%) | 0 (0%) |
| **Relationship to patient**  **Spouse** | 21 (35.0%) | --- |
| **Son/daughter** | 16 (26.7%) | --- |
| **Sibling** | 8 (13.3%) | --- |
| **Parent** | 6 (10.0%) | --- |
| **Friend** | 6 (10.0%) | --- |
| **Cousin** | 2 (3.3%) | --- |
| **Girlfriend/boyfriend** | 1 (1.7%) | --- |
| **Lives in same metropolitan area as the medical center** | 20 (33.3%) | --- |
| **Years worked at hospital** | --- | 11.6 (11.1) |

*Numbers are presented as n (%) or mean (SD) depending on variable type**.**
