# Supplementary material for: Measuring the quality of inpatient specialist consultation in the intensive care unit: Nursing and family experiences of communication
Source: PLoS One. 2019 Apr 11;14(4):e0214918. doi: 10.1371/journal.pone.0214918 (PMC6459595; doi:10.1371/journal.pone.0214918)
Supplement: S4 Table — (DOCX) [file pone.0214918.s004.docx]

Data Supplement for

*“Measuring the quality of inpatient specialist consultation in the intensive care unit: Nursing and family experiences of communication”*

Stephanie D. Roche, Alyse M. Reichheld, Nicholas Demosthenes, Anna C. Johansson, Michael D. Howell, Michael N. Cocchi, Bruce E. Landon, Jennifer P. Stevens

The de-identified dataset is available from the Harvard Dataverse repository at <https://doi.org/10.7910/DVN/JDJBSR>.

**S4 Table. Univariate associations of patient and participant features, divided by participants’ communication with the subspecialist team***

|  |  |  | **Family Members** | |  | | **P Value** |  | **Nurses** | |  | **P Value** |
| --- | --- | --- | --- | --- | --- | --- | --- | --- | --- | --- | --- | --- |
|  |  |  | **Direct Communication**  n=20 | **Limited Communication**  n=40 | |  |  |  | **Direct Communication**  n=85 | **Limited Communication**  n=75 |  |  |
| Participant Characteristics | **Female** |  | 14 (70.0%) | 25 (62.5%) | |  | **0.57** |  | 82 (96.5) | 71 (94.7%) |  | **0.71** |
|  | **Age, mean** |  | 55.3 (12.1) | 54.3 (13.8) | |  | **0.78** |  | 39.1 (12.1) | 38.6 (11.6) |  | **0.81** |
|  | **Age category, years**  18 to 39 |  | 3 (15.0%) | 7 (17.5%) | |  | **1.00** |  | 47 (56.0%) | 47 (63.5%) |  | **0.60** |
|  | 40 to 59 |  | 11 (55.0%) | 20 (50.0%) | |  |  |  | 33 (39.3%) | 25 (33.8%) |  |  |
|  | 60 to 79 |  | 6 (30.0%) | 12 (30.0%) | |  |  |  | 4 (4.8%) | 2 (2.7%) |  |  |
|  | 80 to 99 |  | 0 (0.0%) | 1 (2.5%) | |  |  |  | 0 (0%) | 0 (0%) |  |  |
|  | **Relationship to Patient**  Spouse |  | 7 (35.0%) | 14 (35.0%) | |  | **0.60** |  | --- | --- |  | --- |
|  | Son/daughter |  | 7 (35.0%) | 9 (22.5%) | |  |  |  | --- | --- |  |  |
|  | Sibling |  | 3 (15.0%) | 5 (12.5%) | |  |  |  | --- | --- |  |  |
|  | Parent |  | 2 (10.0%) | 4 (10.0%) | |  |  |  | --- | --- |  |  |
|  | Other |  | 1 (5.0%) | 8 (20.0%) | |  |  |  | --- | --- |  |  |
|  | **Lives in same metropolitan area as the medical center** |  | 7 (35.0%) | 13 (32.5%) | |  | **0.85** |  | --- | --- |  | --- |
|  | **Years at hospital, mean** |  | --- | --- | |  | --- |  | 11.1 (10.7) | 10.4 (10.2) |  | **0.68** |
|  | **Work experience at hospital, category**  Low: <5 years |  | --- | --- | |  | --- | 42 (50.0%) | | 36 (48.7%) |  | **0.20** |
|  | Intermediate: 6-10 years |  | --- | --- | |  |  |  | 8 (9.5%) | 14 (18.9%) |  |  |
|  | High: >10 years |  | --- | --- | |  |  |  | 34 (40.5%) | 24 (32.4%) |  |  |
| Patient Characteristics | **Female** |  | 8 (40.0%) | 16 (40.0%) | |  | **1.00** |  | 34 (40.0%) | 27 (36.0%) |  | **0.60** |
|  | **Age category, years**  18 to 39 |  | 3 (15.0%) | 2 (5.0%) | |  | **0.08** |  | 9 (10.6%) | 5 (6.7%) |  | **0.68** |
|  | 40 to 59 |  | 2 (10.0%) | 13 (32.5%) | |  |  |  | 18 (21.2%) | 19 (25.3%) |  |  |
|  | 60 to 79 |  | 11 (55.0%) | 22 (55.0%) | |  |  |  | 42 (49.4%) | 40 (53.3%) |  |  |
|  | 80 to 99 |  | 4 (20.0%) | 3 (7.5%) | |  |  |  | 16 (18.8%) | 11 (14.7%) |  |  |
|  | **SOFA admit**  0 to 6 |  | 8 (40.0%) | 17 (42.5%) | |  | **0.68** |  | 40 (47.1%) | 31 (41.3%) |  | **0.75** |
|  | 7 to 9 |  | 6 (30.0%) | 15 (37.5%) | |  |  |  | 25 (29.4%) | 19 (25.3%) |  |  |
|  | 10 to 12 |  | 4 (20.0%) | 4 (10.0%) | |  |  |  | 10 (11.8%) | 13 (17.3%) |  |  |
|  | 13 to 14 |  | 2 (10.0%) | 2 (5.0%) | |  |  |  | 4 (4.7%) | 5 (6.7%) |  |  |
|  | 15 to 24 |  | 0 (0%) | 2 (5.0%) | |  |  |  | 6 (7.1%) | 7 (9.3%) |  |  |
|  | **SOFA day of consultation**  0 to 6 |  | 9 (45.0%) | 16 (40.0%) | | **0.97** | |  | 33 (38.8%) | 26 (34.7%) |  | **0.89** |
|  | 7 to 9 |  | 8 (40.0%) | 14 (35.0%) | |  |  |  | 29 (34.1%) | 23 (30.7%) |  |  |
|  | 10 to 12 |  | 2 (10.0%) | 5 (12.5%) | |  |  |  | 13 (15.3%) | 14 (18.7%) |  |  |
|  | 13 to 14 |  | 1 (5.0%) | 3 (7.5%) | |  |  |  | 4 (4.7%) | 5 (6.7%) |  |  |
|  | 15 to 24 |  | 0 (0%) | 2 (5.0%) | |  |  |  | 6 (7.1%) | 7 (9.3%) |  |  |
|  | **LOS, days** |  | 17.0 (13.6) | 17.7 (15.0) | |  | **0.87** |  | 17.8 (14.8) | 16.1 (15.4) |  | **0.46** |
|  | **Consultation type**  Medical |  | 18 (90.0%) | 33 (82.5%) | |  | **0.70** |  | 77 (90.6%) | 63 (84.0%) |  | **0.21** |
|  | Surgical |  | 2 (10.0%) | 7 (17.5%) | |  |  |  | 8 (9.4%) | 12 (16.0%) |  |  |
|  | **Campus - East** |  | 7 (35.0%) | 10 (25.0%) | |  | **0.42** |  | 23 (27.1%) | 20 (26.7%) |  | **0.95** |
|  | **Weekend Consultation** |  | 0 (0%) | 2 (5.0%) | |  | **0.55** |  | 4 (4.7%) | 2 (2.7%) |  | **0.68** |
| Consultation Quality Rating | **Timeliness****  Excellent |  | 15 (79.0%) | 13 (68.4%) | |  | **0.57** |  | --- | --- |  | --- |
|  | Good |  | 3 (15.8%) | 5 (26.3%) | |  |  |  | --- | --- |  |  |
|  | Okay |  | 0 (0%) | 1 (5.3%) | |  |  |  | --- | --- |  |  |
|  | Bad |  | 1 (5.3%) | 0 (0%) | |  |  |  | --- | --- |  |  |
|  | Terrible |  | 0 (0%) | 0 (0%) | |  |  |  | --- | --- |  |  |
|  | **Consultation added value to patient’s care****  Yes |  | --- | --- | |  |  |  | 78 (97.5%) | 44 (86.3%) |  | **0.03** |
|  | No |  | --- | --- | |  |  |  | 2 (2.5%) | 7 (13.7%) |  |  |
|  | **Overall quality of consultation****  Excellent |  | --- | --- | |  | --- |  | 31 (37.3%) | 4 (7.1%) |  | **<.0001** |
|  | Good |  | --- | --- | |  |  |  | 36 (43.4%) | 27 (48.2%) |  |  |
|  | Okay |  | --- | --- | |  |  |  | 15 (18.1%) | 19 (33.9%) |  |  |
|  | Bad |  | --- | --- | |  |  |  | 1 (1.2%) | 6 (10.7%) |  |  |
|  | Terrible |  | --- | --- | |  |  |  | 0 (0%) | 0 (0%) |  |  |

*Numbers are presented as n (%) or mean (SD) depending on variable type and distribution.

**Participants answering “I don’t know/remember” were excluded from these frequency counts.
