# Supplementary material for: Measuring the quality of inpatient specialist consultation in the intensive care unit: Nursing and family experiences of communication
Source: PLoS One. 2019 Apr 11;14(4):e0214918. doi: 10.1371/journal.pone.0214918 (PMC6459595; doi:10.1371/journal.pone.0214918)
Supplement: S5 Table — (DOCX) [file pone.0214918.s005.docx]

Data Supplement for

*“Measuring the quality of inpatient specialist consultation in the intensive care unit: Nursing and family experiences of communication”*

Stephanie D. Roche, Alyse M. Reichheld, Nicholas Demosthenes, Anna C. Johansson, Michael D. Howell, Michael N. Cocchi, Bruce E. Landon, Jennifer P. Stevens

The de-identified dataset is available from the Harvard Dataverse repository at <https://doi.org/10.7910/DVN/JDJBSR>.

**S5 Table. Univariate associations of patient and participant features, divided by participants’ rating of the consultation’s quality***

|  |  |  | **Family Members**** | |  | | **P Value** |  | **Nurses**** | |  | **P Value** |
| --- | --- | --- | --- | --- | --- | --- | --- | --- | --- | --- | --- | --- |
|  |  |  | **Excellent Timeliness Rating**  n=28 | **< Excellent Timeliness Rating**  n=10 |  |  | |  | **Excellent Quality Rating**  n=35 | **< Excellent Quality Rating**  n=104 |  |  |
| Participant Characteristics | **Female** |  | 20 (71.4%) | 7 (70.0%) |  | **1.00** | |  | 34 (97.1) | 99 (95.2%) |  | **0.62** |
|  | **Age, mean** |  | 54.7 (12.8) | 53.5 (15.5) |  | **0.81** | |  | 38.1 (12.0) | 37.8 (11.5) |  | **0.90** |
|  | **Age category, years**  18 to 39 |  | 5 (17.9%) | 1 (10.0%) |  | **0.70** | |  | 19 (55.9%) | 67 (65.05%) |  | **0.34** |
|  | 40 to 59 |  | 16 (57.1%) | 5 (50.0%) |  |  |  |  | 13 (38.2%) | 34 (33.0%) |  |  |
|  | 60 to 79 |  | 6 (21.4%) | 4 (40.0%) |  |  |  |  | 2 (5.9%) | 2 (1.9%) |  |  |
|  | 80 to 99 |  | 1 (3.6%) | 0 (0%) |  |  |  |  | 0 (0%) | 0 (0%) |  |  |
|  | **Relationship to Patient**  Spouse |  | 10 (35.7%) | 3 (30.0%) |  | **0.005** | |  | --- | --- |  | --- |
|  | Son/daughter |  | 12 (42.9%) | 0 (0%) |  |  |  |  | --- | --- |  |  |
|  | Sibling |  | 4 (14.3%) | 2 (20.0%) |  |  |  |  | --- | --- |  |  |
|  | Parent |  | 1 (3.6%) | 1 (10.0%) |  |  |  |  | --- | --- |  |  |
|  | Other |  | 1 (3.6%) | 4 (40.0%) |  |  |  |  | --- | --- |  |  |
|  | **Lives in same metropolitan area as the medical center** |  | 9 (32.1%) | 3 (30.0%) |  | **1.00** | |  | --- | --- |  | --- |
|  | **Years at hospital, category**  <5 years |  | --- | --- |  | --- | | 20 (58.8%) | | 52 (50.5%) |  | **0.55** |
|  | 6-10 years |  | --- | --- |  |  |  |  | 3 (8.8%) | 16 (15.5%) |  |  |
|  | >10 years |  | --- | --- |  |  |  |  | 11 (32.4%) | 35 (34.0%) |  |  |
| Patient/Consultation Characteristics | **Female** |  | 10 (35.7%) | 3 (30.0%) |  | **1.00** | |  | 15 (42.9%) | 41 (39.4%) |  | **0.72** |
|  | **Age categories**  18 to 39 |  | 2 (7.1%) | 2 (20.0%) |  | **0.31** | |  | 4 (11.4%) | 9 (8.7%) |  | **0.73** |
|  | 40 to 59 |  | 4 (14.3%) | 2 (20.0%) |  |  |  |  | 6 (17.1%) | 26 (25.0%) |  |  |
|  | 60 to 79 |  | 16 (57.1%) | 6 (60.0%) |  |  |  |  | 18 (51.4%) | 53 (51.0%) |  |  |
|  | 80 to 99 |  | 6 (21.4%) | 0 (0%) |  |  |  |  | 7 (20.0%) | 16 (15.4%) |  |  |
|  | **SOFA admit**  0 to 6 |  | 12 (42.9%) | 5 (50.0%) |  | **1.00** | |  | 13 (37.1%) | 40 (38.5%) |  | **0.49** |
|  | 7 to 9 |  | 9 (32.1%) | 4 (40.0%) |  |  |  |  | 8 (22.9%) | 37 (35.6%) |  |  |
|  | 10 to 12 |  | 5 (17.9%) | 1 (10.0%) |  |  |  |  | 9 (25.7%) | 15 (14.4%) |  |  |
|  | 13 to 14 |  | 2 (7.1%) | 0 (0%) |  |  |  |  | 2 (5.7%) | 5 (4.8%) |  |  |
|  | 15 to 24 |  | 0 (0%) | 0 (0%) |  |  |  |  | 3 (8.6%) | 7 (6.7%) |  |  |
|  | **SOFA day of consultation**  0 to 6 |  | 13 (46.4%) | 5 (50.0%) | **0.97** | | |  | 14 (40.0%) | 36 (34.6%) |  | **0.25** |
|  | 7 to 9 |  | 11 (39.3%) | 4 (40.0%) |  |  |  |  | 9 (25.7%) | 37 (35.6%) |  |  |
|  | 10 to 12 |  | 2 (7.1%) | 1 (10.0%) |  |  |  |  | 8 (22.9%) | 14 (13.5%) |  |  |
|  | 13 to 14 |  | 2 (7.1%) | 0 (0%) |  |  |  |  | 0 (0%) | 8 (7.7%) |  |  |
|  | 15 to 24 |  | 0 (0%) | 0 (0%) |  |  |  |  | 4 (11.4%) | 9 (8.7%) |  |  |
|  | **LOS, days** |  | 17.4 (14.2) | 14.8 (8.6) |  | **0.59** | |  | 15.3 (13.5) | 17.1 (15.2) |  | **0.55** |
|  | **Consultation type**  Medical |  | 24 (85.7%) | 8 (80.0%) |  | **0.64** | |  | 33 (94.3%) | 89 (85.6%) |  | **0.24** |
|  | Surgical |  | 4 (14.3%) | 2 (20.0%) |  |  |  |  | 2 (5.7%) | 15 (14.4%) |  |  |
|  | **Campus - East** |  | 11 (39.3%) | 2 (20.0%) |  | **0.44** | |  | 15 (42.9%) | 20 (19.2%) |  | **0.005** |
|  | **Weekend Consultation** |  | 0 (0%) | 0 (0%) |  | **--** | |  | 2 (5.7%) | 4 (3.9%) |  | **0.64** |
|  | **Type of Communication**  Direct communication |  | 15 (53.6%) | 4 (40.0%) |  | **0.46** | |  | 31 (88.6%) | 52 (50.0%) |  | **<.0001** |
|  | No direct communication |  | 13 (46.4%) | 6 (60.0%) |  |  |  |  | 4 (11.4%) | 52 (50.0%) |  |  |

*** Numbers are presented as n (%) or mean (SD) depending on variable type and distribution.

**Only 38 of 60 (63%) families felt that they knew enough about the consultation to offer an assessment of its timeliness. In 139 of 160 (87%) consultations, the nurse felt s/he could offer an assessment of the consultation’s overall quality. All numbers are presented as n (%) or mean (SD) depending on variable type and distribution.
